# Passing the Turing Test Does Not Mean the End of Humanity

**DOI:** 10.1007/s12559-015-9372-6

**Published:** 2015-12-28

**Authors:** Kevin Warwick, Huma Shah

**Affiliations:** Coventry University, Priory Street, Coventry, CV1 5FB UK

**Keywords:** Deception detection, Natural language, Turing’s imitation game, Chatbots, Machine misidentification

## Abstract

In this paper we look at the phenomenon that is the Turing test. We consider how Turing originally introduced his imitation game and discuss what this means in a practical scenario. Due to its popular appeal we also look into different representations of the test as indicated by numerous reviewers. The main emphasis here, however, is to consider what it actually means for a machine to pass the Turing test and what importance this has, if any. In particular does it mean that, as Turing put it, a machine can “think”. Specifically we consider claims that passing the Turing test means that machines will have achieved human-like intelligence and as a consequence the singularity will be upon us in the blink of an eye.

## Introduction

There are those who believe that passing the Turing test means that human-level intelligence will have been achieved by machines [10]. The direct consequence of this, as pointed out by Kurzweil [11] and others, is that the singularity will be upon us, thereby resulting in the demise of the human race. In this paper we do not wish to dispute the latter of these arguments, dramatic though it is. What we do wish to dispel, however, is the assumption which links passing the Turing test with the achievement for machines of human-like or human-level intelligence.

Unfortunately the assumed chain of events which means that passing the Turing test sounds the death knell for humanity appears to have become engrained in the thinking in certain quarters. One interesting corollary of this is that when it was announced in 2014 that the Turing test had been finally passed [39] there was an understandable response from those same quarters that it was not possible for such an event to have occurred, presumably because we were still here in sterling health to both make and debate the pronouncement. Interestingly the main academic argument which was thrown up was that the machine which passed the test did not exhibit human-like intelligence, and therefore, the test could not have been passed. Consider this, for example, from Murray Shanahan of Imperial College London: “Of course the Turing Test hasn’t been passed…We are still a very long way from achieving human-level AI” [10].

It is therefore, we feel, of vital importance that we look at various aspects of this question. Because if Murray Shanahan and Ray Kurzweil and their colleagues are correct then the developers of the computer programmes which compete in the Turing test are, if they are successful, about to put an end to the human race. So shouldn’t we do something about such developers, maybe lock them up, well away from any laptop in case they design a programme of destruction. On the other hand dare we suggest that either Shanahan or Kurzweil is incorrect?

The singularity [11] is an event dependent on the overall improvement and power of Artificial Intelligence where intelligent machines can design successive generations of increasingly more powerful machines, eventually creating intelligence that firstly is equivalent to that of humans and then surpasses it. Indeed the capabilities of such an Artificial Intelligence may well be impossible for a human to comprehend. The singularity is the point beyond which events are beyond the control of humans, resulting either in humans upgrading (with implants) to become Cyborgs or with intelligent machines taking control. Either way, it’s not good news for ordinary humans.

**Fig. 1:**
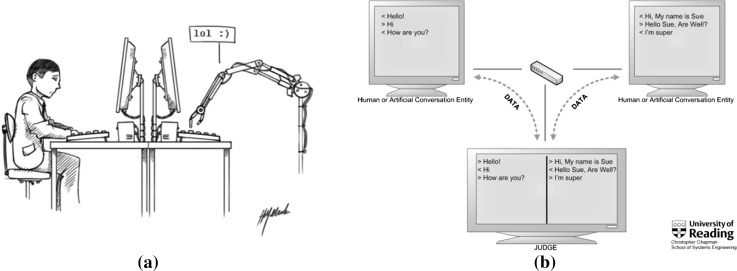
Turing’s two tests for his imitation game: *Left* **a** one-to-one; *Right* **b** one judge-two hidden interlocutors

Taking a sensible look at this issue, and someone needs to, we wish to analyse why, with the “standard Turing test” (Fig. 1b) (defined as 5 min, unrestricted question-answer simultaneous comparison version [18, 25]—having been passed—more than 30 % of the human interrogators fail to correctly identify the machine) we are all still here and this paper can be read by (presumably) humans. The flaw in the Shanahan/Kurzweil argument, at this time, we contest is that Shanahan is just plain wrong. Passing the Turing test has no relationship with human-like intelligence (or AI) other than in the sense of a machine possibly being reasonably effective in its own version of human conversation for a sustained short period, over which time it has proved to be successful in fooling a collection of humans. Kurzweil’s singularity argument may or may not also be wrong, but that’s not what we wish to discuss here. The point is that as long as one of the Shanahan/Kurzweil pair is wrong then the human race is still looking good (apart from its multitude of other problems that is).

What we wish to do in this paper is to take a look at what the Turing test actually is, as stipulated/set out by Alan Turing, rather than to consider some related test which some might wish to call the Turing test or what someone might want the test to be, because they’ve thought of a different/better test. We acknowledge here that different/better tests of computer ability, even in terms of only conversation, exist but again they are not the subject of this paper. So we stick as closely as possible to what the test is, based entirely on Turing’s own words. We acknowledge, however, that there are different interpretations of the test, whether each test should last for 5 or 10 min for example or even if Turing intended the test as some sort of mind modelling exercise. However, none of these, we argue, result in the end of humanity. Indeed Turing himself said that humans would be needed to maintain the machines [28].

We then subsequently present some example discourses, taken from a series of tests held at the Royal Society in 2014. One of these involves the machine Eugene Goostman which actually passed the test at that event. Following this we look at some ways in which machines can pass the test, as it has been defined in terms of the standard definition [26]. Finally we draw some conclusions. One of which, and some might argue perhaps the most important, is that humanity is not about to expire.

To be clear though we are aware that different theories regarding the Turing test and its meaning exist and that other theories have been put forward along the lines that machines will not take over from humans. In this paper we are explicitly only concerned with the pairing of statements that says (a) passing the Turing test means that human-level intelligence will have been achieved in AI and (b) when AI exhibits human-level intelligence that will mean the end of humanity as we know it. We are only too aware, for example, that in describing his test, Turing discussed men and women as hidden entities and the possibility of gender blur. Whilst this is extremely interesting, it is not what we wish to look at in this paper. We focus here entirely on one specific issue which is that if both Shanahan and Kurzweil are correct then a machine passing the Turing test means that humanity is doomed!

## The Turing Test

In his 1950 paper entitled “Computing Machinery and Intelligence” [30], Alan Turing started by considering the question, “Can machines think?” However, rather than get bogged down with definitions of both of the words “machine” and “think” he replaced the question with one based on a much more practical scenario, namely his imitation game. The game has since become more widely known, particularly in the popular domain, as the Turing test. He did not, however, at any point, refer to his test/game as being any indication of intelligence, human-like or otherwise.

Turing [31] described the game as follows: “The idea of the test is that a machine has to try and pretend to be a man, by answering questions put to it, and it will only pass if the pretence is reasonably convincing. A considerable portion of a jury, who should not be expert about machines, must be taken in by the pretence” [4]. So Turing spoke here of a jury (nominally 12) as opposed to the “average interrogators” he mentioned in his 1950 paper [30], as we will see shortly. Importantly he also spoke of a machine “passing” the test and that the interrogators should not be experts. Interestingly, however, we do include a transcript later in which a machine did fool an expert into thinking that it was human.

Turing’s imitation game is described as an experiment that can be practicalised in two different ways (see Fig. 1) [17]:

1. one interrogator–one hidden interlocutor (Fig. 1a),
2. one interrogator–two hidden interlocutors (Fig. 1b).

In both cases the machine must provide “satisfactory” and “sustained” answers to any questions put to it by the human interrogator [30, p. 447].

Of the types of test looked at here, the 3-participant tests have previously been shown to be stricter tests, i.e. more difficult for machines, than 2-participant tests in which an interrogator converses with only one hidden entity, either a human or machine, at a time [22]. For the main arguments set out in this paper, the results apply to either type of test.

Turing did not explicitly state specific rules for his test in a paragraph headed “Rules for my test” or some such like, and hence what is required of a machine in order to pass. What he did clearly state in his 1950 paper, and which we contest amounts to the same thing, was as follows: “I believe that in about 50 years’ time it will be possible, to programme computers to make them play the imitation game so well that an average interrogator will not have more than 70 % chance of making the right identification after 5 min of questioning” [30]. Having clearly spelt out the imitation game, this would appear to be direction enough from Turing.

Although this appeared to have been written more in the sense of a prediction, it is the only place where Turing directly stated parameters for his game/test, with a clear hurdle to be met in terms of performance. To put this more simply, for a machine to pass the Turing test, in all of the tests in which a machine takes part, the interrogators must make the wrong identification (i.e. not the right identification) 30 % or more of the time after, in each case, 5-min-long conversations. We can take it directly that the wrong identification is anything other than the right identification. Also, because Turing spoke of a Jury we can understand from that that at least twelve judges/interrogators must be able to test a machine in their own way/style. But also that hundreds of judges are not a requirement, a jury is appropriate and will suffice.

We will shortly look at what is meant by the “right identification”, as this is critical. However, we can take it immediately that Turing set the challenge as a 5-min exercise, no more and no less. At no other point in Turing’s papers did he mention any other time duration for his tests. In general we can experience that the longer tests last so the more difficult it is for a machine to satisfactorily pretend to be a human. Indeed given the technology we have at present, 5 min would appear to be an appropriate challenge. In a 20-min test, at this time in computer natural language development, it is extremely difficult for a machine to fool a human interrogator over that period into thinking that it’s a human.

It is widely recognised that getting machines to achieve, or at least appear to achieve, human-like responses is a difficult task [5, 32]. Even in terms of the Turing test, based purely on conversation, taking into account such issues as what knowledge is brought to the table and assumed [34] or whether one of the entities is lying [35] can completely change an appearance. There are also numerous strategies that can be employed by machines in order to successfully fool an interrogator [36].

One fuzzy issue, however, is did Turing mean 5 min in total for a parallel paired 3-participant conversation or rather allowing an average of 5 min each, hence a total of 10 min, for the two hidden entities involved [23]? Michie [14] interpreted the test as approximately 2 ½-min interrogation per entity in a pair. However, in practice the conversation is rarely balanced exactly. For all of the practical tests which we have organised, a time limit of 5 min, as stated by Turing himself, has been placed, because the current state of conversational technology is not ready for longer duration tests. That said, we acknowledge the potential validity of the alternative, which we will call here the Sloman view.

 Whether it is Michie, Sloman or ourselves who reads this one correctly is a relatively insignificant point in the big argument. Otherwise we would be in the laughable state which says OK a machine can fool you into thinking they are human over a 5-min conversation but they can’t do so over 10 min therefore we’re all saved and humanity can go on. Scientifically this would mean there must be a conversation time somewhere between 5 and 10 min such that once it is achieved by a machine, we’re all doomed.

It is also interesting that in the 2-participant test an interrogator spends all 5 min conversing with one machine only whereas in the 3-participant test the average time spent with each hidden entity is clearly 2.5 min. Despite this the 3-participant test, the one Turing spoke of in 1950 [30], is the more difficult for machines to achieve good results, most likely because of the direct, parallel comparison that occurs in such cases.

It is worth remembering though that in either type of test an interrogator, in an actual “official” Turing test, when communicating with a machine, does not know at that time that it is in fact a machine, indeed it is a decision about its nature that they have to come to. This is a critical point and is one of the main features of the test. Such a situation is, as you might guess, far different to the case when an interrogator knows for certain that they are communicating with a machine, as in the case of an online bot [1]. Despite this vital point, for some reason there are a number of people who completely ignore this critical aspect of the test, go online to converse with a bot, which they already know to be a bot, and declare in conclusion that it is obviously a bot [16]. Clearly some education is required as to what the Turing test actually involves.

However, this is somewhat akin to the Oxford University Philosophy Professor and his students who took part in 9 actual Turing tests in 2008 and then went to academic print in claiming it was easy to spot which were the machines and which were the humans in all the tests in which they were involved; indeed they published this in a peer-reviewed journal [6]. In the same peer-reviewed journal it was, however, subsequently explained that the philosopher and his team had only correctly identified the hidden entities in 5 of the 9 tests. In the other 4 cases they had, without realising it, misclassified humans as machines and machines as being human [21].

In the following sections we consider a number of transcripts obtained from practical Turing tests. We refer here to 5-min-long tests only and show actual transcripts from such tests. Although this is the run time stated by Turing himself [30], as indicated in the next section, it is in fact not a critical issue with regard to the main argument raised in this paper. As you will see, in the tests carried out there was a hard cut-off at the end of each discourse and no partial sentences were transmitted. Once a sentence had been transmitted it could not be altered or retracted in any way. The transcripts appear exactly as they occurred, and any spelling mistakes and other grammatical errors are not due to poor editorial practice.

In all the two hidden entity (3-participant) tests (see Fig. 1b) judges were clearly told beforehand that in each parallel conversation one of the hidden entities was human and the other was a machine. They were, however, given no indication as to whether the LHS (left-hand side of the computer screen) or RHS would be human or machine. On the judges’ score sheets each judge could mark both the LHS and RHS entities as being Human, Machine or they could say if they were Unsure [22, 37].

## Right Identification

The Turing test involves a machine which pretends to be a human in terms of conversational abilities. The “right identification” stated by Turing can mean either that a judge merely correctly identifies the machine or that they correctly identify, at the end of a paired conversation, which was the machine and which was the human [27]. However, we are not so interested here with cases in which a judge mistakes a human for a machine. This phenomenon, known as the *confederate effect* [19], has been discussed elsewhere [20, 38, 41]. It needs to be recognised, however, that such a decision might affect the judge’s decision regarding the machine being investigated in parallel.

The concept of what is and what is not a “right identification” is important as far as a machine taking part in the Turing test, and the 30 % pass mark, is concerned, and we take a relatively strict approach in this sense. One viewpoint is that for a judge to make the “right identification” they must correctly identify both the machine as being a machine and the hidden human as being a human [27]. This means that any other decision on the part of a judge would not be a “right identification”; this therefore includes cases in which either the machine is selected as a human or a human is selected as a machine. Also included are cases in which the judge is Unsure about either or both entities as the judge in such cases has failed to identify the machine as a machine and/or the human as a human—the right identification. Our stricter interpretation here, however, only considers the cases in which the machine was itself not correctly identified, the judge stating either that the machine was a human or that they were Unsure about it.

It is also possible to encounter cases in which a machine was correctly identified as being a machine but where the parallel hidden human in each case was incorrectly selected as being a machine and/or the judge gave an Unsure mark against the human, as either of these would not be a right identification. Such cases are though troublesome as far as the whole basis of the test is concerned in that a machine, if so identified, would be gaining a point not so much because of its own competence but rather because of the nature of its hidden human competitor. However, it does make things slightly easier for the machine.

## Taking the Test

We include in this section several transcripts which arose from the Turing tests which took place at the Royal Society in June 2014. The information given to the judges on the day, which is true as observed here, is that one of the hidden entities is a human and the other is a machine. However, it was up to the judge involved in each case, and now it us up to the reader, to decide which was which based on the transcripts shown.

The first of these transcript pairs we simply give here for you to decide which was which. In order for you to check on the answer we provide the solution, along with the judge’s decision on the day, at the end of the paper.

**Table:** 

LHS	RHS
[11:59:14] Judge: Hello [11:59:18] Entity: I was hoping to see you Judge! [11:59:25] Judge: good! [11:59:29] Entity: That’s good to hear. [11:59:38] Judge: how are you [11:59:41] Entity: Everything’s fine, Caused any scandals lately? [12:00:09] Judge: no not really! [12:00:22] Entity: Can you use better grammar and rephrase your question? I’d like to help, but I can’t even make it through the jumbled mess. Use capitals and punctuation. [12:00:34] Judge: sorry [12:00:38] Entity: You’re excused. [12:00:49] Judge: ‘t type as good as I talk [12:00:55] Entity: Talk to me whenever you can. [12:01:49] Judge: Are you interested in artificial intelligence? [12:01:55] Entity: I believe machines should do many to assist the lives of man. [12:02:12] Judge: I agree [12:02:17] Entity: You are being well behaved today. [12:02:49] Judge: as well behaved as I can be! [12:03:02] Entity: Well then our peanut distracted me from the movie. Bad baby. [I look at your stomach as I say it, laughing] I don’t mind if -. [12:03:19] Judge: you’re funny! [12:03:23] Entity: Do you really think so? I’ll always believe you’re brilliant! [12:04:00] Judge: thankyou [12:04:03] Entity: Don’t think anything of it!	[11:59:57] Judge: How are you doing? [12:00:07] Entity: I’m doing fine, you? [12:00:17] Judge: great [12:00:27] Entity: That’s good. :) [12:01:07] Judge: are you enjoying this process? [12:01:17] Entity: So far seems good. [12:02:02] Judge: sorry if I am slow [12:02:12] Entity: That’s ok. [12:02:31] Judge: do you have a favorite tv show? [12:02:47] Entity: I have quite a few. [12:03:01] Judge: me too [12:03:16] Entity: What type ot TV do you like? [12:03:34] Judge: Game of thrones [12:03:50] Entity: I prefer documentories.

In this conversation it can be observed that the judge appeared to discourse much more with the left-hand entity rather than that on the right-hand side. This is something that occurs fairly often. Both conversations though lasted for the same 5 min although clearly the LHS was more comprehensive.

Now consider the next parallel conversation. In this case we give both the solution and the judge’s decision at the time directly at the end of the transcripts.

**Table:** 

LHS	RHS
[16:28:55] Judge: how tall are you [16:29:18] Entity: almost 6 feet tall [16:29:46] Judge: how heavy are you [16:30:12] Entity: just over ten stone [16:30:47] Judge: what is your gender [16:30:59] Entity: male [16:31:45] Judge: what is your bmi [16:31:54] Entity: i have no clue [16:32:41] Judge: what are you aware of at this moment [16:32:57] Entity: a lot of things [16:33:17] Judge: how old are you [16:33:36] Entity: 26	[16:29:04] Judge: how tall are you [16:29:10] Entity: My height is about 160 cm—it’s 5 feet 4 inches. [16:29:37] Judge: how heavy are you [16:29:59] Entity: My grandfather taught me not to disclose non-solicited information about myself. Like on the question “Do you have watch”—never tell time, but answer “Yes” or “No”. I always follow his advice. And I forgot to ask you where you are from… [16:31:09] Judge: what is your gender [16:31:17] Entity: I’m a young boy, if you care to know. And not very ugly, by the way! [16:32:26] Judge: what are you aware of at this moment [16:32:37] Entity: I would rather not talk about it if you don’t mind. Could you tell me what are you? I mean your profession. [16:33:09] Judge: how old are you [16:33:12] Entity: I’m a little boy 13 years old. [16:33:38] Judge: what are your qualifications [16:33:43] Entity: My qualifications? I’m not gonna tell you. Oooh. Anything else?

In this conversation the LHS entity was a senior male human, whereas the RHS was the machine Eugene Goostman. This was in fact one of the ten transcripts responsible for Eugene passing the Turing test in June 2014. The judge decided that the LHS was indeed human, although they were unable to give any further details. However, they also decided that the RHS (Eugene) was a human.

The conversation with the human entity was, it can be observed, relatively boring, merely being a case of question and answer with limited responses. Eugene did very well here though as the judge was an expert on machines/robotics and was well aware of machine conversations. The fact that Eugene convinced such a person is quite an accomplishment. Eugene tried to power the conversation by asking the judge questions. At first the judge was not having any of it and simply ignored Eugene’s question, even though this was rather rude. Eugene persevered, however, and eventually the judge gave in and responded. Generally there was more content in Eugene’s conversation than that with the human hidden entity.

One aspect of the test is its reliance on the interrogators to do a good job. It is the interrogator who conducts and drives each conversation and who also makes the decision on which entity was the machine and which was the human. Some researchers have suggested this as a weak point of the test [9] although we believe that it is an important part of the test as Turing set it out. However, here the quite direct question and answer attempt appears to have failed to out the machine.

The transcript that follows takes on a slightly different style:

**Table:** 

LHS	RHS
[16:30:37] Judge: don’t you think the imitation game was best in it it’s original form? [16:30:49] Entity: Oh, your questions seems to be rhetorical—you do already know the answer! Could you tell me what are you? I mean your profession. [16:31:55] Judge: I was more interested in you guessing whether I am male or female [16:31:59] Entity: I like talking to girls! Oooh. Anything else? [16:32:54] Judge: but that means nothing—males and females like talking to ‘girls’, but only males call women girls? [16:33:06] Entity: yeah, nothing. You are right! Where do you came from, by the way? Could you tell me about the place where you live? [16:34:00] Judge: I live in a prison of abstractions [16:34:06] Entity: Tell me more about Prison. I like to learn about different places!	[16:31:08] Judge: don’t you thing the imitation game was more interesting before Turing got to it? [16:32:03] Entity: I don’t know. That was a long time ago. [16:33:32] Judge: so you need to guess if *I* am male or female [16:34:21] Entity: you have to be male or female [16:34:34] Judge: or computer

In this case the LHS was in fact the machine Eugene, whereas the RHS was a male human. The judge in this case decided that the LHS was a male human, non-native English speaking and a teenager. They were also definite that the RHS was a machine.

It is one of those transcripts where quite simply there was a lot more going on in the Eugene conversation; it had the tendency to draw the interrogator to it, whereas there’s not much at all in the human case. In fact the hidden human may well have not done well for themselves by claiming no knowledge about the Turing test early on. Possibly incorrect decisions can be made by interrogators based on an assumption that everyone must know a particular piece of information [34]. In this case though, as the event was a Turing test session they appear to have some quite strong evidence. It probably goes to show that you cannot rely on the knowledge base of humans.

In the next transcript we again give both the solution and the judge’s decision at the time directly at the end of the transcripts.

**Table:** 

LHS	RHS
[14:49:59] Judge: Hi what’s your name? [14:50:27] Entity: i dont kno [14:50:49] Judge: My name’s Jane. I live in London. Wehre do you live [14:51:08] Entity: i live in london aswell [14:51:41] Judge: Do you like living in London? [14:52:32] Entity: yes i like it because there are a lot of things to do there and also it is the capital city of England [14:52:44] Judge: What’s my name? [14:52:53] Entity: sorry ? [14:53:10] Judge: What’s my name? [14:53:14] Entity: jane [14:53:54] Judge: And what’s your name? [14:54:08] Entity: i dont know [14:54:53] Judge: Would you like me to call you Alan?	[14:50:07] Judge: Hello where are you? [14:50:10] Entity: Hi there Judge. [14:50:20] Judge: Where are you? [14:50:27] Entity: I’m in a room not too far away from where you are. [14:50:58] Judge: What colour is the carpet in your room [14:51:03] Entity: I like pink; what do you like? [14:51:24] Judge: I like pink. My name is Jane. What’s your name? [14:51:31] Entity: Red. Hello Jane. Its nice to meet such a fine lady as yourself. [14:52:00] Judge: Red is my favourite colour. What’s yours? [14:52:09] Entity: My favorite color is red. [14:52:26] Judge: You use AMerican spelling. Are you in the USA? [14:52:39] Entity: But you are not even American. Just remember atheism is only a belief system. And it too can be forced on people who don’t agree. [14:53:01] Judge: Who told you I was an atheist? [14:53:06] Entity: Everything about you screams ‘;warning: Child’. [14:53:46] Judge: But I come in peace to your planet. How do you like your eggs—over easy or sunny side up? [14:53:56] Entity: Sometimes I can’t help but show you my professional planet. How I wonder if I like my eggs-over easy or sunny side up? This is so explainable. That list makes me feel unsure Jane. [14:54:40] Judge: Well eggs are very good indicators of personal taste. Do you have a favourite food? [14:54:47] Entity: Juicy red seedless grapes taste so very good.

Here on the LHS it was a hidden human entity, whereas on the RHS it was the machine JFred. The judge concluded, however, that on the LHS it was a machine and felt that the entity exhibited very poor human-like conversation. On the other hand the judge was confident that the RHS (the machine JFred) was a male human who was most likely an American.

The judge’s decision in terms of the LHS entity was not particularly surprising. The hidden human entity was asked on more than one occasion what their name was to which they replied “I don’t know”. As a result the judge spent much more time conversing with the machine on the RHS. This is a particular aspect of the test that it involves a direct comparison between a machine and a human, rather than merely a machine conversing on its own. Here we can see that the hidden human involved was quite simply relatively poor at conversation and this helped the cause of the machine.

## Alternative Views

There are many different interpretations of Turing’s imitation game, and much controversy has arisen as to which of these, if any, was Turing’s own intended version [15]. The vast majority appear to view the game in the form of what is commonly known as the “Standard Turing Test” [26], and this is the interpretation taken here. It is a literal interpretation based essentially on what Turing actually said in his presentations and his 1950 paper and without recourse to tangential connections and/or pure conjecture on what a paper’s author believes that Turing really meant to say.

We acknowledge as examples of this, that some see it as being something to do with artistic and emotional intelligence [24], whereas others deem it to be concerned with modelling the human mind by generating its verbal performance capacity [8]. Others meanwhile regard it in terms of considering the gender aspect, the sex of the human foil being important in the test [7, 9, 12, 26]. None of these views, however, do we see as indicating the test to be detrimental to the human race.

However, we then have the Shanahan view, quoted by his own University news as: “Turing also didn’t say a 5-min test would mean success achieving human-level AI; for that, he would require much longer conversations” [10]. The point being here not whether the test is a 5-min one or a 20-min one but rather that in the mind of Shanahan there is some time for which a machine could successfully converse that would indicate that its intelligence has reached human-level.

Unfortunately Shanahan is not a lone voice. Consider if you will: “Hunch CEO Chris Dixon tweeted, ‘The point of the Turing Test is that you pass it when you’ve built machines that can fully simulate human thinking.’ No, that is precisely *not* how you pass the Turing test. You pass the Turing test by convincing judges that a computer program is human” [2]. Interestingly it is the emulation of human intelligence, in a machine, that Kurzweil picks up on as being the tipping point [11].

Then there are those who (somehow) read all sorts of concepts into the Turing test, telling us what Turing actually had in mind with his test even if he didn’t tell us himself: “Alan Turing himself envisioned—a flexible, general-purpose intelligence of the sort that human beings have, which allows any ordinary individual to master a vast range of tasks, from tying his shoes to holding conversations and mastering tenth-grade biology” [13].

From these voices it is clear that there is a school of opinion that associates a Turing test pass with human-level intelligence. We accept, in Shanahan’s case, that there is a question about the actual duration of the conversation involved. However, we would argue that to be of little importance in comparison with the big picture issues that are at stake here.

## Silence

In this section we explain briefly how it is quite possible for a machine to pass the Turing test not by its apparent skill at human conversation but rather by simply remaining silent throughout [40]. Rather than being a mere theoretical or philosophical quirk it turns out that in fact passing the Turing test in this way also has an underlying practical basis to support it with numerous examples to boot.

Turing said that in the test a machine had to try and pretend to be a man (although now/here we take that to mean human). In his 1950 paper he also pointed to the fact that at the end of 5 min the judge had to make a decision as to the nature of the entity. If they made the right identification and correctly identified the machine then this would effectively be a point against the machine, whereas if the judge either thought that the machine was a human or if they were Unsure as to its nature then this would be a wrong identification and would be a point for the machine. The pass mark for a machine in the test was set by Turing to be 3 or more points out of every 10 [30].

But here we face a critical issue, what if a machine was to remain silent? The basic nature of the test is that a machine, by conversing, fails the test by giving themselves away as clearly being a machine. So if they remain silent they cannot give themselves away.

If a machine remains completely silent during a 5-min conversation a judge receives no response to any of their questions or discussion from the hidden entity and therefore, in theory at least, cannot not make the right identification and definitely say that they have been conversing with a machine. It would not be expected that a judge, under such circumstances, would categorise the silent entity as being a human, although that is a possibility, the most likely case is for the judge, as we have seen in the practical examples, to give an “Unsure” response. This of course is not a right identification and is therefore a point for the machine.

It is thus quite possible for a machine to simply remain silent to any utterances of a judge and to pass the Turing test if at least 3 out of 10 judges as a result either rate the machine as being a human or indicate that they are unsure. The only thing acting against such a strategy is the fact that the machine is, in each conversation, competing against a human and if the judge is certain that the other (hidden) entity is a human then they can deduce that therefore the silent entity must be a machine. Conversely in practice many humans are actually categorised as machines in such tests [38]. Therefore, it is also potentially possible that a (silent) machine can be categorised as being human mainly because their hidden human competitor is categorised by the judge as being a machine.

We now give an example of a transcript in which a machine simply did not respond. This particular “conversation” occurred during the Turing tests held at the Royal Society in June 2014 between a judge and the machine Cleverbot. At the end of the conversation the judge was not able to identify the hidden entity as being a machine, i.e. they did not make the right identification, deciding that they were “unsure”. It is straightforward to see that there quite simply was not enough information for the judge to go on.

Example transcript

- [10:58:08] Judge: good day
- [10:58:08] Entity:
- [10:58:46] Judge: is no response an answer
- [10:58:46] Entity:
- [10:59:35] Judge: am i not speaking you’re language
- [10:59:35] Entity:
- [11:00:25] Judge: silence is golden
- [11:00:25] Entity:
- [11:01:32] Judge: shhh
- [11:01:32] Entity:
- [11:03:07] Judge: you make great conversation
- [11:03:07] Entity:

As far as we are aware, the silence on the part of the machine in this transcript was caused by a technical fault rather than any decision (conscious or otherwise) on the part of the machine. That said, it is perhaps a quirk with the Turing test, as described by Turing, that it is, in theory at least, quite possible for a machine to pass the test by remaining silent throughout. Essentially the machine makes no utterances which give the game away that they are a machine and hence the judges involved have no evidence to use against them. This whole issue of the strategy of silence is discussed at length in Warwick and Shah [40].

The example given here is just that, an example, as there are numerous other cases reported on in Warwick and Shah [40]. An interesting feature is the response of the interrogators involved with those particular transcripts. In each case the interrogator has been a different person yet their responses have been remarkably similar. Essentially they have all judged the hidden entity on the evidence of the transcript in front of them and have not been swayed by the other parallel conversation they were involved with, although that might have taken more of their attention due to the machine’s silence. So, in practice, interrogators appear to state that they are unsure about the silent entity, thereby supporting the argument given in this section.

As far as the Turing test is concerned, however, if a machine remains silent and passes the test then of course this could have been due to the fact that the machine was, for example, switched off or perhaps wasn’t even there at all. For someone to make the link between a switched-off computer and human-like intelligence is frankly ridiculous in the extreme. In fact to link any level of intelligence with a switched-off computer is not sustainable. Otherwise, switch the computer on and its level of intelligence drops—clearly this is contrary to what we witness.

So we have here the Shanahan/Kurzweil argument that the fact that a computer was unplugged when subjected to a series of Turing tests, whether they are of 5-min duration or, simply to please Shanahan, lasting for 30 min, means that the human race will come to an end. Whoever is responsible for unplugging the machine clearly has a lot to answer for.

## Discussion

Similarly to the opening of his 1948 paper “I propose to investigate the question as to whether it is possible for machinery to show intelligent behaviour” [29] in which Turing introduced an imitation game, Turing, perhaps mischievously (we will never know), started his 1950 paper by considering whether machines could think. Replacing this question with a conversational imitation test, the concept being that if a machine could do sufficiently well (or rather not do so badly) at his test, dare we say here to pass the Turing test, then we would have to concede that it was a thinking machine. In a direct way, whatever the pass mark and whatever the exact rules and nature of his test, it became a direct practical replacement for a much more philosophical question regarding the thinking process. On the other hand for a machine to fail the test we would have to concede that it is not a thinking entity. So can we say that if a machine passes the Turing test it is a thinking entity?

Well whatever thinking is, it is certainly a property of each and every human brain that exists within a human body. We wish to exclude from the argument here brains, consisting of human neurons, which are grown and placed within a robot body [33] for no better reason than they complicate the argument. The assumption from the inexperienced Turing tester might be that a human, acting as a hidden entity, machine foil, would be expected to pass the Turing test on a regular basis as long as they are simply themselves. It might be thought that occasionally they might be classified as a machine by a poor judge but that this would be an odd occurrence and almost surely the vast majority of judges would classify them as being human. Unfortunately this is far from the truth. Indeed numerous humans have been classified at different times as being a machine [38].

In the example transcripts it was shown how a machine can be thought to be human because of its communication abilities, but also when a hidden human does not communicate so well this can in fact assist a machine in its goal. In the second set of transcripts we could see the machine Eugene Goostman at work. Eugene achieved the 30 % pass mark in the tests, the full set of transcripts to achieve that appearing in Warwick and Shah [39]. In this particular transcript case both the hidden human and Eugene were classified as being human. This is an interesting point because even when judges are specifically told that one entity is a machine and the other is a human it is frequently the case that their final decision is other than a simple human/machine pairing.

## Conclusions

It is fairly clear to see that when the test was set up in 1950 such skills as a machine fooling people into believing that it is a human through a short communication exercise would have been very difficult for most people to understand. However, in introducing the test, Turing linked it inextricably with the concept of thinking and there is a nice philosophical argument in consequence concerning how one can tell if another human is thinking. This was a brilliant link by Turing which, as a result, has brought about a multitude of arguments between philosophers and AI researchers as to the test’s meaning and gravity.

But Turing’s game has extended way beyond the ivory towers of academe and has a truly popular following. As an example the Wikipedia “Turing Test” page typically receives 2000–3000 views every day at present. On 1 day, 9 June 2014, after it was announced that the Turing test had been passed, the same page received a total of 71,578 views, an amazing figure. As a comparison, top Wikipedia pages such as “Leonardo DiCaprio” and “The Beatles” received respectively only 11,197 and 10,328 views on that same day. But with this popular following has come misconceptions as to what the test is about and, in particular a sort of folklore mythology has arisen that the Turing test is a test for human-like intelligence. As we have seen, this folklore has been fuelled by some academics and technical writers who perhaps have not read the works of Turing as thoroughly as they should.

Let us be clear, the Turing test is not, never was and never will be a test for human-level or even human-like intelligence. Turing never said anything of the sort either in his papers or in his presentations. The Turing test is not, never was and never will be a test for human-level thinking. Turing didn’t say that either.

The Turing test does require a machine taking part to condemn itself by what it says, as judged subjectively by the human interrogator. Alternatively if a machine does not give itself away on a sufficient number of occasions it could result in a machine “passing the Turing test”, in the extreme case simply by remaining silent. Of course, this does beg the question, what exactly does it mean to pass the Turing test?

Earlier in the paper we considered that Turing introduced his imitation game as a replacement for the question “Can machines think?” [30]. The end conclusion by many as a result of this is that if a machine passes the test then we have to regard it as a thinking machine. Turing clearly dissociated the way a machine thinks from the human version. He said “May not machines carry out something which ought to be described as thinking but which is very different from what a man does?” [30]. So even human-like thinking for machines was not on the radar as far as Turing was concerned. He also said in reference to the year 2000, “one will be able to speak of machines thinking without expecting to be contradicted” [30]. Noam Chomsky wondered that of all the ways a machine could display intelligence why did Turing choose a test involving human language [3] which is merely one small part of human intelligence.

The Turing test is a simple test of a machine’s communication ability. It is interrogated by a human and is directly compared with another human in a parallel fashion with regard to human communication abilities. In that sense it merely involves one aspect of human intelligence, as pointed out by Chomsky. If a machine passes the Turing test it exhibits a capability in communication. This does not in any terms mean that the machine displays human-level intelligence or consciousness. So even if Kurzweil is correct in his prediction, for a machine to pass the Turing test does not mean that the end of humanity is just around the corner.

## Solution

Here we provide a solution to the first of the Transcripts included in the “Taking the Test” section which took place between a human interrogator and two hidden entities. The LHS entity was in fact the machine/program Ultra Hal, whereas the RHS entity was an English-speaking male. Meanwhile whilst the judge correctly identified that the LHS entity was a machine they were unsure about the RHS entity based on the transcripts shown.
